# Genomic Variants Among Threatened *Acropora* Corals

**DOI:** 10.1534/g3.119.400125

**Published:** 2019-03-26

**Authors:** Sheila. A. Kitchen, Aakrosh Ratan, Oscar C. Bedoya-Reina, Richard Burhans, Nicole D. Fogarty, Webb Miller, Iliana B. Baums

**Affiliations:** * 208 Mueller Lab, Biology Department; ** Centre for Comparative Genomics and Bioinformatics, The Pennsylvania State University, University Park PA 16802; † Department of Public Health Sciences and Center for Public Health Genomics, University of Virginia, Charlottesville VA 22908; ‡ MRC Functional Genomics Unit, Department of Physiology, Anatomy and Genetics, University of Oxford, South Parks Road, Oxford OX1 3PT, UK; § MRC Human Genetics Unit, MRC Institute of Genetics and Molecular Medicine, The University of Edinburgh, Western General Hospital, Crewe Road, Edinburgh, UK, and; †† Department of Marine and Environmental Sciences, Nova Southeastern University, Fort Lauderdale, FL 33314

**Keywords:** coral, Caribbean, single nucleotide polymorphism, population genomics, Galaxy

## Abstract

Genomic sequence data for non-model organisms are increasingly available requiring the development of efficient and reproducible workflows. Here, we develop the first genomic resources and reproducible workflows for two threatened members of the reef-building coral genus *Acropora*. We generated genomic sequence data from multiple samples of the Caribbean *A. cervicornis* (staghorn coral) and *A. palmata* (elkhorn coral), and predicted millions of nucleotide variants among these two species and the Pacific *A. digitifera*. A subset of predicted nucleotide variants were verified using restriction length polymorphism assays and proved useful in distinguishing the two Caribbean acroporids and the hybrid they form (“*A. prolifera*”). Nucleotide variants are freely available from the Galaxy server (usegalaxy.org), and can be analyzed there with computational tools and stored workflows that require only an internet browser. We describe these data and some of the analysis tools, concentrating on fixed differences between *A. cervicornis* and *A. palmata*. In particular, we found that fixed amino acid differences between these two species were enriched in proteins associated with development, cellular stress response, and the host’s interactions with associated microbes, for instance in the ABC transporters and superoxide dismutase. Identified candidate genes may underlie functional differences in how these threatened species respond to changing environments. Users can expand the presented analyses easily by adding genomic data from additional species, as they become available.

Genomic data for non-model organisms are becoming available at an unprecedented rate. Analyses of these data will advance our understanding of the capacity of organisms to adapt, acclimatize or shift their ranges in response to rapid environmental change (Savolainen *et al.* 2013). While genome sequencing itself has become routine, bioinformatics treatment of the data still presents hurdles to the efficient and reproducible use of this data (Nekrutenko and Taylor 2012). Thus, genomic variant analysis workflows (*e.g.*, Bedoya-Reina *et al.* (2013)) are needed to eliminate some of these computational hurdles and increase reproducibility of analyses. Here, we develop such tools, apply them to threatened reef-building corals, and present novel findings with respect to the molecular pathways used by these species to respond to environmental stimuli.

The *Acropora* species, *A. cervicornis* and *A. palmata* were the main reef-building corals of the Caribbean (Figure 1). These corals have greatly decreased in abundance during recent years due to infectious disease outbreaks, habitat degradation, storm damage, coral bleaching, outbreaks of predators, and anthropogenic activities (Bruckner 2002). A large body of previous studies has investigated the effects of environmental stress in Caribbean acroporid corals (Randall and Szmant 2009; DeSalvo *et al.* 2010; Baums *et al.* 2013; Libro *et al.* 2013; Polato *et al.* 2013; Parkinson *et al.* 2015). These studies highlight changes in the molecular, cellular, and physiological response of these species to an unprecedented elevation in seawater temperature. Increases in water temperature of only 2-3° can reduce the fertilization rates, reduce larval survival, and deplete genotypic diversity of Caribbean acroporids (Randall and Szmant 2009; Williams and Miller 2012; Baums *et al.* 2013).

**Figure 1:**
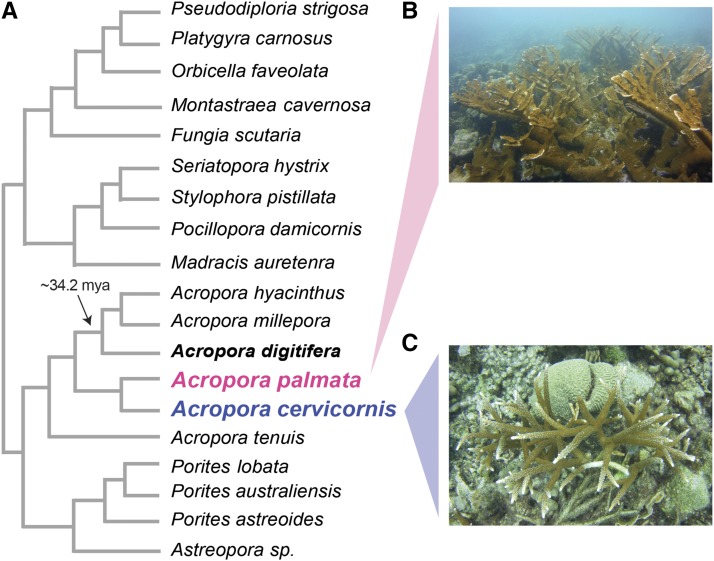
Phylogeny of corals with genomic and transcriptomic resources used in this study (A) with images of the two focal species, *Acropora palmata* (B) and *Acropora cervicornis* (C). The evolutionary relationships depicted in the coral phylogeny are redrawn based on the phylogenomic analysis by Bhattacharya *et al.* (2016), but branch lengths do not reflect evolutionary distance. Estimate of divergence time between the Caribbean acroporids and *A. digitifera* was calculated by Richards *et al.* (2013). Photographs of *A. palmata* (B) and *A. cervicornis* (C) were taken by Iliana B. Baums in Curacao (2018).

Because of a tremendous die-off, both species are now listed as threatened on the United States Federal Endangered Species List (Anonymous 2006). Extensive conservation efforts are currently underway across the range, which will be considerably facilitated by the acquisition of genomic data. For instance, these data will help to identify management units, evolutionary significant units, hybridization dynamics, genotypic diversity cold-spots and interactions with the corals’ obligate symbionts in the genus *Symbiodinium* (Baums 2008; van Oppen *et al.* 2015). The project described here represents an early effort to move beyond low-resolution sequencing and microsatellite studies (Vollmer and Palumbi 2007; Baums *et al.* 2014) and employ the power of full-genome analysis (Drury *et al.* 2016).

Here, we present genome-wide single nucleotide variants (SNVs) between the two Caribbean acroporids relying on the genome assembly for a closely related species, *A. digitifera* (Shinzato *et al.* 2011) (Figure 1). We have successfully used the same approach to analyze genomes of *Apis mellifera* populations (Fuller *et al.* 2015) to much more distant reference species, such as polar, brown, and black bears based on the dog genome (Miller *et al.* 2012), and giraffe based on cow and dog genomes (Agaba *et al.* 2016). We highlight several examples of how these SNVs enable population genomic and evolutionary analyses of two reef-building coral species. The SNV results are available on the open source, public server Galaxy (Afgan *et al.* 2016), along with executable histories of the computational tools and their settings. This workflow presented here for corals and by Bedoya-Reina *et al.* (2013) can be transferred for genomic analyses of other non-model organisms and provide abundant information in a reproducible manner.

## Materials and Methods

### DNA Extraction and Sequencing

For each species, five previously genotyped samples from the Baums Lab coral tissue collection were selected from each of the four sites representing their geographic range: Florida (FL), Belize (BE), Curacao (CU) and U.S. Virgin Islands (VI; Table 1) (Baums *et al.* 2009; Baums *et al.* 2005). An additional sample for each species from Florida (*A. cervicornis* CFL14120 and *A. palmata* PFL1012) was selected for deep genome sequencing because they are located at easily accessible and protected sites in the Florida Keys (*A. palmata* at Horseshoe Reef and *A. cervicornis* at the Coral Restoration Foundation nursery) and are predictable spawners that are highly fecund. High molecular weight DNA was isolated from each sample using the Qiagen DNeasy kit (Qiagen, Valencia, CA) according to the manufacturer’s protocol. DNA quality and quantity was assessed with gel electrophoresis and Qubit 2.0 fluorometry (Thermo Fisher, Waltham, MA), respectively. Sequence library construction and sequencing was completed by the Pennsylvania State University Genomics Core Facility. Paired-end short insert (550 nt) sequencing libraries of the two deeply sequenced genomes were constructed with 1.8-2 µg sample DNA and the TruSeq DNA PCR-Free kit (Illumina, San Diego, CA). The remaining 40 paired-end short insert (350 nt) sequencing libraries (Table S1) were constructed using 100 ng sample DNA and the TruSeq DNA Nano kit (Illumina, San Diego, CA). Deep- and shallow-sequence libraries were pooled separately and sequenced on the Illumina HiSeq 2500 Rapid Run (Illumina, San Diego, CA) over two lanes and four lanes, respectively.

**Table 1:** Sequenced Genomes. Species assignment was based initially on microsatellite multilocus genotyping. *Acropora* Genet ID is an identifier for each *Acropora* multilocus microsatellite genotype in the Baums Lab database. Coordinates are given in decimal degrees (WGS84). Two samples were sequenced to a greater depth (bold type)

Species	Region	Sample ID	*Acropora* Genet ID	Reef	Latitude	Longitude	Collection Date	SRA Accession
***A. cervicornis***	Belize	CBE13827	C1630	Glovers Atoll	16.88806	−87.75973	8-Nov-15	SRR7236033
		CBE13837	C1631	Glovers Atoll	16.88806	−87.75973	8-Nov-15	SRR7236028
		CBE13792	C1632	Sandbores	16.77913	−88.11755	7-Nov-15	SRR7236031
		CBE13797	C1646	Sandbores	16.77913	−88.11755	7-Nov-15	SRR7236034
		CBE13786	C1569	South Carrie Bow Cay	16.80132	−88.0825	6-Nov-15	SRR7236032
	Curacao	CCU13917	C1648	Directors Bay	12.066	−68.85997	4-Feb-16	SRR7236036
		CCU13925	C1649	East Point	12.04069	−68.78301	5-Feb-16	SRR7235996
		CCU13901	C1647	SeaAquarium	12.0842	−68.8966	2-Feb-16	SRR7236030
		CCU13903	C1650	SeaAquarium	12.0842	−68.8966	2-Feb-16	SRR7236029
		CCU13905	C1651	SeaAquarium	12.0842	−68.8966	2-Feb-16	SRR7236037
	Florida	CFL4927	C1471	CRF	25.2155	−80.60778	22-Nov-11	SRR7235993
		CFL4959	C1476	CRF	24.9225	−81.12417	22-Nov-11	SRR7235991
		CFL4923	C1484	CRF	25.16472	−80.59389	22-Nov-11	SRR7235994
		CFL4928	C1485	CRF	25.03222	−80.50417	22-Nov-11	SRR7235992
		**CFL14120**	**C1297**	**CRF (Grassy Key)**	**24.71182**	**−80.94595**	**1-Mar-16**	**SRR7235995**
		CFL4960	C1297	CRF (Grassy Key)	24.71182	−80.94595	22-Nov-11	SRR7235990
	USVI	CVI13712	C1633	Botany	18.3569	−65.03515	28-Oct-15	SRR7235999
		CVI13696	C1638	Botany	18.3569	−65.03515	27-Oct-15	SRR7235989
		CVI13758	C1456	Flat Key	18.31701	−64.9892	31-Oct-15	SRR7236022
		CVI13714	C1644	Hans Lollik	18.40191	−64.9063	29-Oct-15	SRR7235998
		CVI13738	C1628	Sapphire	18.3333	−64.8499	30-Oct-15	SRR7236021
***A. palmata***	Belize	PBE13813	P2947	Glovers Atoll	16.88806	−87.75973	8-Nov-15	SRR7236017
		PBE13819	P2959	Glovers Atoll	16.88806	−87.75973	8-Nov-15	SRR7236015
		PBE13801	P2964	Sandbores	16.77913	−88.11755	7-Nov-15	SRR7236020
		PBE13784	P2945	South Carrie Bow Cay	16.80132	−88.0825	5-Nov-15	SRR7236019
		PBE13815	P2951	South Carrie Bow Cay	16.80132	−88.0825	5-Nov-15	SRR7236018
	Curacao	PCU13919	P2970	Directors Bay	12.066	−68.85998	4-Feb-16	SRR7235988
		PCU13933	P2977	East Point	12.04069	−68.78301	5-Feb-16	SRR7235987
		PCU13911	P1232	SeaAquarium	12.0842	−68.8966	3-Feb-16	SRR7235985
		PCU13907	P2212	SeaAquarium	12.0842	−68.8966	3-Feb-16	SRR7235986
		PCU13939	P2976	Water Factory	12.1085	−68.9528	6-Feb-16	SRR7235982
	Florida	PFL5524	P2118	Carysfort	25.22178	−80.2106	1-Aug-05	SRR7236012
		PFL2655	P1032	Elbow	25.14363	−80.25793	3-Jun-10	SRR7235979
		PFL2699	P2564	French	25.03393	−80.34941	28-May-10	SRR7236011
		**PFL1012**	**P1000**	**Horseshoe**	**25.13947**	**−80.29435**	**25-Apr-01**	**SRR7235983**
		PFL1037	P1001	Little Grecian	25.11843	−80.31715	2-Jul-02	SRR7235980
		PFL6895	P1003	Sand Island	25.01817	−80.36832	17-Sep-09	SRR7236001
	USVI	PVI13702	P2957	Botany	18.3569	−65.03515	27-Oct-15	SRR7236003
		PVI13752	P2946	Flat Key	18.31701	−64.9892	31-Oct-15	SRR7236010
		PVI13744	P2953	Hans Lollik	18.40191	−64.9063	29-Oct-15	SRR7236008
		PVI13750	P2954	Hans Lollik	18.40191	−64.9063	29-Oct-15	SRR7236009
		PVI13740	P2952	Sapphire	18.3333	−64.8499	30-Oct-15	SRR7236007

### A. digitifera Assembly and Inter-species Gene Model Comparisons

We downloaded the *A. digitifera* genome assembly and GFF-formatted gene annotations from NCBI (GCA_000222465.2 Adig_1.1). To conduct the pathway enrichment analysis, we obtained additional annotation from the Kyoto Encyclopedia of Genes and Genomes (KEGG) (Kanehisa *et al.* 2017). During gene prediction, gene annotation can be error prone and misled by assembly gaps or errors, imprecision of *de novo* gene predictors and/or errors in gene annotations in the species used for comparison, among other sources. To overcome these known issues, our approach included, at a minimum, submitting the putative amino acid sequence to the blastp server maintained by the Reef Genomics Organization (Liew *et al.* 2016) (http://comparative.reefgenomics.org/blast/) and the blastp and/or psi-blast servers at NCBI (Altschul *et al.* 1997) (http://blast.ncbi.nlm.nih.gov). We also used the Reef Genomics website to assess the degree of inter-species sequence conservation among 20 corals in Figure 1 (resources include transcriptomes and genomes, details provided in Bhattacharya *et al.* (2016), and the Genome Browser (http://genome.ucsc.edu) at the University of California at Santa Cruz (Kent *et al.* 2002)) to measure the inter-species conservation of the orthologous mammalian residue. We interpret the degree of conservation at a protein position and its immediate neighbors as suggesting the amount of selective pressure and the functional importance of the site.

### Single Nucleotide Variant and Indel Calls

We aligned the paired-end sequences for the 42 samples to the *A*. *digitifera* reference genome sequence using BWA version 0.7.12 (Li and Durbin 2009) with default parameters. On average, we were able to align ∼89% of the reads for each individual, and ∼74% of the reads aligned with a mapping quality > 0. Paired-end reads are generated by sequencing from both ends of the DNA fragments, and we found that about 70% of these reads aligned within the expected distance from its mate in those alignments (see Table S1 for details). We used SAMBLASTER version 0.1.22 (Faust and Hall 2014) to flag potential PCR duplicate reads that could otherwise affect the quality of the variant calls (Table S1). Considering data from all individuals simultaneously, we used SAMtools version 1.3.1 (Li *et al.* 2009) to identify the locations of putative variants with parameters –g to compute genotype likelihoods, -A to include all read pairs in variant calling, and –E to recalculate the base alignment quality score against the reference *A. digitifera* genome. Variants were called with bcftools version 1.2 (Li 2011) multiallelic caller and further filtered to keep those variants for which the total coverage in the samples was less than 1,200 reads (to limit the erroneous calling of variant positions in repetitive or duplicated regions), the average mapping quality was greater than 30, and the fraction of reads that aligned with a zero mapping quality was less than 0.05. The VCF file of nucleotide variants was converted to gd_snp format using the “Convert” tool from the “Genome Diversity” repository on Galaxy, after separating the substitution and insertion/deletion (indel) variants. The mitochondrial variants were similarly identified using the *A. digitifera* mitochondrial reference genome (GenBank: NC_022830), and variant locations were drawn using the Python program Millerplot (https://github.com/aakrosh/Millerplot).

The Galaxy tool “Phylogenetic Tree” under Genome Diversity (Bedoya-Reina *et al.* 2013) was used to calculate the genetic distance between two individuals at a given SNV based on their genotype call. For instance, if the two genotypes are 2 and 1, *i.e.*, the samples are estimated to have respectively 2 and 1 occurrences of the first allele at this location, then the distance is 1 (the absolute value of the difference of the two numbers). This is repeated for all SNV to calculate the overall genetic distance matrix. The Neighbor-joining tree was constructed with QuickTree (Howe *et al.* 2002) and visualized with draw_tree utility script in package PHAST (Hubisz *et al.* 2010). We compared this simplistic phylogenetic approach to an identity-by-state analysis using the R package SNPRelate (Zheng *et al.* 2012). We used I-TASSER online server for protein structure prediction (Yang *et al.* 2015) to model and further help to develop hypotheses about functionality of several mutations in STE20-related kinase adapter protein alpha protein (NCBI: LOC107340566) and phosphatidylcholine translocator ABCB4-like protein (NCBI: LOC107340542). Identification of enriched KEGG pathways was completed using the “Rank Pathways” tool, which compares the gene set with SNVs against the complete set of genes in the pathway using the statistical Fisher’s exact test. The Galaxy tool does not include a multiple test correction, therefore a randomization analysis was completed by simulating 1,000 gene sets of the same size as the target list and calculating the expected number of false discoveries (*i.e.*, average number of hypothesis rejected in the random sets) and the FDR for various uncorrected p-value thresholds. This procedure indicated that enriched KEGG pathways with uncorrected p-values of ≤ 0.01 are not false discoveries.

### Genomic Regions of Differentiation

*F*_ST_ values can be used to find genomic regions where the two species have allele frequencies that are remarkably different over a given window or interval, *i.e.*, the *F*_ST_ values are unusually high. Such intervals may indicate the location of a past “selective sweep” (Akey *et al.* 2002) caused by a random mutation that introduces an advantageous allele, which rises to prominence in the species because of selective pressures, thereby increasing the frequency of nearby variants and changing allele frequencies from those in an initially similar species. In theory, the *F*_ST_ ranges between 0, when the allele frequencies are identical in the two species, to 1, for a fixed difference. However, in practice it works better to use an estimation formula that accounts for the limited allele sampling; we employ the “unbiased estimator” of Reich *et al.* (2009) because it performs best on the kinds of data used here, according to Willing *et al.* (2012). While the *F*_ST_ provides a relative estimate of genomic difference between two populations, calculation of pairwise differences between sequences from two populations excluding within-population polymorphisms provides a measurement of absolute divergence (d_XY_) (Nei 1987). In this way, d_XY_ represents the accumulation of mutations within a genomic interval since the split from the most recent common ancestor (Cruickshank and Hahn 2014).

To identify regions of differentiation, individuals were assigned to their respective species using the “Specify Individuals” tool and then a summary of the allele calls for all individuals was calculated using the “Aggregate individuals” tool. We assigned a measure of allele frequency difference to each SNV analogous to calculations of *F*_ST_ for intra-species comparisons using the “Remarkable Intervals” Galaxy tool (score shift set to 90%, (Bedoya-Reina *et al.* 2013) based on the results of the “Per-SNP FSTs” tool using the Reich-Patterson estimator. Intervals of high scoring SNVs were identified by subtracting 0.90 from each SNV *F*_ST_ value and totaling the score of consecutive SNVs until the score could no longer be increased by the addition or subtraction of one or more SNVs on either end. It should be noted that care must be taken when interpreting high *F*_ST_ values this way, since they can also be caused by genetic drift, demographic effects, or admixture (Holsinger and Weir 2009).We compared these intervals to the genome-wide *F*_ST_ estimate calculated using the Galaxy tool “Overall FST”.

Nucleotide diversity (π) and d_XY_ were estimated from the high-scoring *F*_ST_ intervals or a comparable number of random intervals from the filtered high quality SNVs in 500 bp non-overlapping windows with at least 10 SNVs per window using the Python script popgenWindows.py (https://github.com/simonhmartin/genomics_general, (Martin *et al.* 2015)).

### PCR-Ready SNV Markers and RFLP Validation

PCR-ready SNVs were identified based on the following criteria: 1) the SNV-caller considered them to be high-quality (Phred-scaled quality score ≥ 900), 2) all 21 *A. cervicornis* samples looked homozygous for one allele while all 21 *A. palmata* samples looked homozygous for the other allele and 3) there were no observed SNVs, indels, low-complexity DNA or unassembled regions within 50 bp on either side of the SNV.

From the PCR-ready SNVs, we developed a PCR-restriction fragment length polymorphism (RFLP) assay to validate a subset of fixed SNVs with additional Caribbean acroporids samples, including the hybrid of the two species, *A. prolifera* (Table S2). We screened 197 fixed SNVs with 50bp flanking sequence (101bp total) using the webserver SNP-RFLPing2 (Chang *et al.* 2006; Chang *et al.* 2010) to find a set of loci that would cut with common restriction enzymes (*Hae*III, *Dpn*II, *Hinf*I, *Eco*RV, and *Hpy*CH4IV all from New England Biolabs, Ipswich, MA). Eight loci were selected, of which half cut *A. palmata*-like SNVs while the other half cut *A. cervicornis*-like SNVs (Table S3). For each diagnostic locus, additional flanking sequence was extracted from the scaffold until another restriction enzyme recognition site was encountered for that specific locus-restriction enzyme combination. Primers were designed for the extended flanking sequence using Primer3web version 4.1.0 (Untergasser *et al.* 2012).

A reference set of parental (*n*= 10 *A. palmata* and *n*= 9 *A. cervicornis*) and hybrid (*n* = 27 colonies) samples from across the geographic range were tested with a previously developed microsatellite assay based on five markers (Baums *et al.* 2005) and the RFLP assay (Table S2). A test set of hybrids (*n* = 20 colonies) that did not have previous genetic information was also included to compare taxon assignment between the two marker sets. Hybrids were initially identified in the field based on intermediate morphological features following Cairns (1982), Oppen *et al.* (2000) and Vollmer and Palumbi (2002).

For all samples, DNA was extracted using the DNeasy kit (Qiagen, Valencia, CA). PCR reactions consisted of 1X NH_4_ Buffer (Bioline, Boston, MA), 3 mM MgCl_2_ (Bioline, Boston, MA), 1 mM dNTP (Bioline, Boston, MA), 250 nmol forward and reverse primers (IDT, Coralville, Iowa), 1 unit of Biolase DNA polymerase (Bioline, Boston, MA) and 1 µl of DNA template for a total volume of 10µl. The profile for the PCR run was as follows: 94° for 4 min for initial denaturing, followed by 35 cycles of 94° for 20s, 55° for 20s, and 72° for 30s, and a final extension at 72° for 30min. For each locus, 5 µl of PCR product was combined with 1X restriction enzyme buffer (New England Biolabs, Ipswich, MA) and 0.2 µl restriction enzyme (New England Biolabs, Ipswich, MA) for a total reaction volume of 10 µl and incubated according to the manufacturer’s recommendation. PCR and digest fragment products were resolved by 2% TAE agarose gel electrophoresis at 100 V for 35 min, except for locus NW_015441368.1: 282878 that was run on 3.5% TAE agarose gel at 75 V for 45 min to resolve the smaller fragments. Banding patterns were scored for each locus as homozygous for either parent species (1 or 2 bands) or heterozygous (3 bands).

Reference samples were first assigned to taxonomic groups (*A. palmata*, *A. cervicornis*, F1 or later generation hybrid) based on allele frequencies at five microsatellite loci (Baums *et al.* 2005) by NEWHYBRIDS (Anderson and Thompson 2002). The group ‘later generation hybrid’ is defined as anything other than F1 hybrids in this study. A discriminant factorial correspondence analysis (DFCA) was performed on the microsatellite and SNV marker data separately to predict sample membership to the taxonomic groups: *A. palmata*, *A. cervicornis*, F1 hybrid or later generation hybrid. The DFCA performed in GENETIX version 4.05 (Belkhir *et al.* 2004) clustered the individuals in multi-dimensional space based on their alleles for each marker type. The factorial axes reveal the variability in the data set with the first factor being the combination of alleles that accounts for the largest amount of variability. The DFCA scores for all axes were used in a two-step discriminant analysis using the R statistical software (RCoreTeam 2017) to calculate the group centroid, or mean discriminant score for a given group, and individual probability of membership to a given group using leave-one-out cross-validation (R code provided in File S1). First, the parameter estimates for the discriminant function of each group were trained by the DFCA scores from the reference samples. Second, those functions were used to assign all samples, including the test set of hybrids, based on their DFCA scores to a taxon group.

### Data Availability

The executable histories for the SNV and protein analyses and their respective data sets are available on Galaxy (https://usegalaxy.org/u/webb/p/coral). The SNV and indel calls for both the nuclear and mitochondrial genomes are available at the Galaxy internet server (https://usegalaxy.org/u/webb/p/coral). Table 2 lists the data sets available on Galaxy. Specifically, the data sets “coral snps” and “intra-codon variants” are tables of variants with positions in reference to the *A. digitifera* genome. The data set “PCR-Ready SNVs” are 101 bp sequences extracted from the *A. digitifera* genome, with 50 bp flanking sequence surrounding the fixed SNV. Raw sequence data are deposited in the NCBI Sequence Read Archive (accessions SRR7235977-SRR7236038). Supplemental figures and tables are uploaded on GSA Figshare (https://gsajournals.figshare.com/s/05a86249b23a046b089c). The R code used to perform the DFCA and generate Figure 5 is provided in File S1. Table S1 is the alignment summary statistics for all samples. Table S2 is the discriminant factorial correspondence analysis results for the microsatellite and SNV markers. Table S3 provides the location, SNV, primers and enzymes for the SNV markers and Table S4 provides their gene annotation. Table S5 is the summary of the gene models identified in the two highest scoring genomic intervals. Figure S1 is the genome coverage of the 21 *Acropora cervicornis* samples. Figure S2 is a phylogenetic tree and identity-by-state analysis of the *Acropora* samples based on high-quality SNVs. Figure S3 presents the locations of mitochondrial variants. Figures S4- S6 and S10 are protein alignments highlighting variants between corals and human ortholog. Figure S7 is an image of the sequence coverage of the 12-bp deletion of STRADα protein. Figure S8 and S9 are protein models of STRADα and ABCB1, respectively. Figure S11 highlights the conservation in ATP-binding cassette sub-family D member 2 in vertebrates. Figure S12 is a comparison of the nucleotide diversity, and relative and absolute divergence between the two species for the genomic islands of divergence. Figure S13 is a gel electrophoresis of RFLP results for two fixed SNV loci. Supplemental material available at https://doi.org/10.25387/g3.7871780.

**Table 2:** Data sets available on Galaxy

Name	Contents	# of Lines
**SNVs**	*A. digitifera* scaffold positions with two observed nucleotides among the three *Acropora* genomes	8,368,985
**indels**	positions and contents of observed short (≤ 20 bp) insertion/deletions	940,345
**SAPs**	protein sequence positions of non-synonymous and synonymous substitutions	561,015
**mitochondrial SNVs**	*A. digitifera* mitochondrial genome positions with two observed nucleotides	172
**mitochondrial indels**	position of an insertion/deletion	1
**exons**	scaffold positions of annotated exon endpoints	222,156
**PCR-ready SNVs**	SNVs where no other SNV, indel, or low-complexity sequence is within 50 bp	894

## Results

### Variants Between Three Acroporid Species

**Figure 2:**
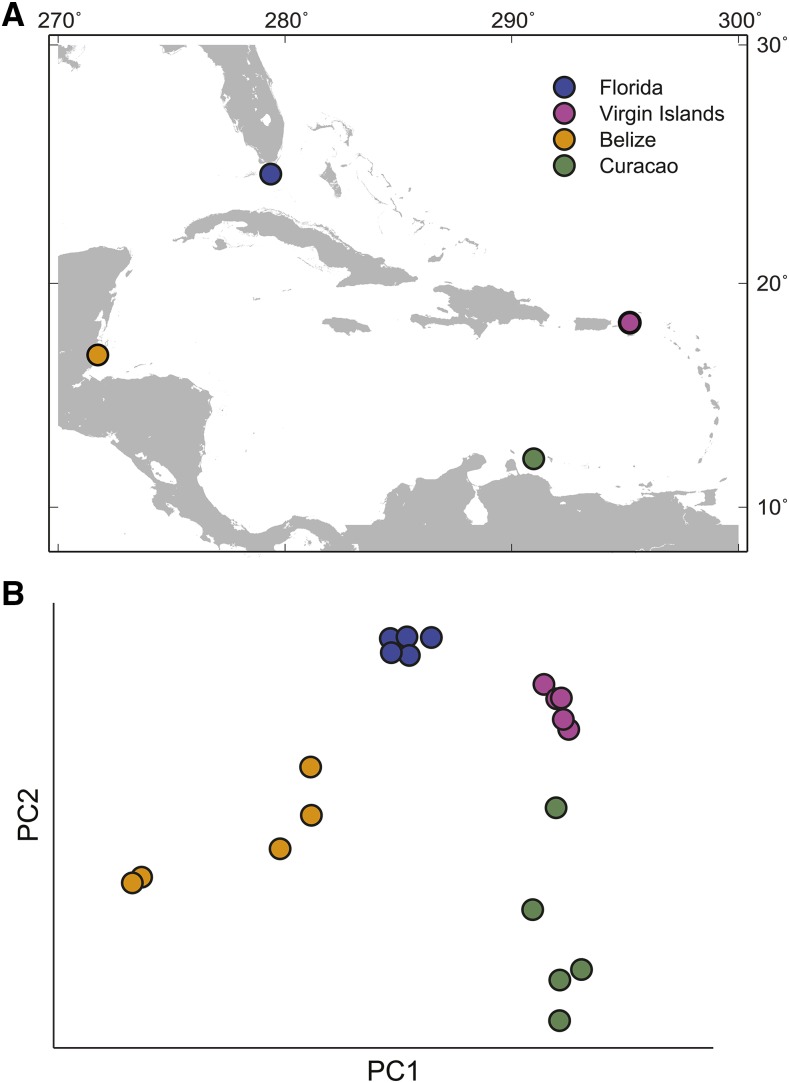
Geographic origin of *Acropora* samples (A) and Principal Components Analysis of *A. cervicornis* samples, five from each of the four locations (B). As noted in analyses of other datasets (*e.g.*, Novembre *et al.* (2008)) the geographic map is similar to the PCA.

For each species, we performed deep-coverage sequencing (roughly 150-fold coverage) of one sample and shallow sequencing (roughly fivefold to 10-fold) of 20 samples, five each from four geographic locations (Florida, the U.S. Virgin Islands, Belize, and Curacao) (Figure 2A). For details, see Table 1. The sequence coverage distribution for the acroporid samples was comparable between species (*A. cervicornis*: Figure S1 and *A. palmata*: “coral SNPs” history at https://usegalaxy.org/u/webb/p/coral).

Rather than relying on *de novo* assembly and gene annotation of our data, we based the analysis reported below on an assembly and annotation of the highly similar reference genome of *A. digitifera* (NCBI: GCA_000222465.2 Adig_1.1) (Shinzato *et al.* 2011). This strategy increases reproducibility and leverages the work of large and experienced bioinformatics groups. Important advantages of using this third species is that we can transfer its gene annotation as well as “polarize” variants. The two sequenced species in this study diverged in the Eocene about 34.2 mya from the most recent common ancestor they share with the reference species *A. digitifera* (Figure 1) (van Oppen *et al.* 2001; Richards *et al.* 2013). Thus, with a difference observed among the *A. cervicornis* and *A. palmata* samples, the allele agreeing with *A. digitifera* can be interpreted as ancestral, and the variant allele as derived.

We identified both substitution and indel variants by aligning our paired-end sequencing reads to the *A. digitifera* assembly and noting nucleotide differences with *A. cervicornis* and *A. palmata* (Table 2). Specifically, each reported substitution variant is a position in an *A. digitifera* assembly scaffold where at least one of our sequenced samples has a nucleotide that is different from the *A. digitifera* reference nucleotide, after all the thresholds on read-depth and mapping quality as discussed in the Methods were applied. We call each of these an SNV (single-nucleotide variant) because “SNP” (single-nucleotide polymorphism) is commonly used to describe an intra-species polymorphism. These data permit comparisons among the three *Acropora* species, although this paper focuses on *A. cervicornis* and *A. palmata*, and ignores unanimous differences of the new sequences from the reference.

### Fixed differences of SNVs and Indels between A. cervicornis and A. palmata

Single nucleotide variants and indels can be used to explore either intra- or inter-species variation, using similar techniques in both cases. Of the 8,368,985 SNVs, 4,998,005 are identically fixed in *A. cervicornis* and *A. palmata*, leaving 3,370,980 variable within our two sequenced species, only 1,692,739 of which were considered high-quality (Phred-scaled quality ≥ 900, Table 2). The results reported below use this set of substitution variants. A phylogenetic tree based on the genetic distance between those SNVs clearly separates the two species, and distinguishes the samples from each species according to where they were collected in most cases (Figure S2A). This relatedness pattern is also reflected in an identity-by-state analysis that calculates the genome-wide average of shared SNVs between samples from the genotype calls (Figure S2B). The same is true of a Principal Component Analysis (Figure 2). From all the SNVs, both synonymous and non-synonymous amino acid substitutions were identified from the coding sequences (Table 2). Out of the 561,015 putative protein-coding SNVs, we retained the 120,206 deemed “high quality” and variable in the two newly sequenced species. To complete our analysis, we identified 172 mitochondrial SNVs, which are highly concentrated in the gene-free “control region” (Figure S3). This region also contains the only identified indel between *A. digitifera* and the two Caribbean acroporids (Figure S3).

The examples in most of the following sections investigate only inter-species differences, and in particular focus on fixed SNVs, *i.e.*, locations where the 21 sequenced *A. cervicornis* samples share the same nucleotide and the 21 *A. palmata* samples share a different nucleotide. Variants were filtered so that the genotype of each shallow genome within a species would match its deeply sequenced genome. This approach identified 65,533 fixed nucleotide SNV differences and 3,256 fixed amino acid differences, spread across 1,386 genes (Table 2, see Galaxy histories “coral SNPs” and “coral proteins”). These SNVs are potentially useful for investigating the genetic causes of phenotypic differences between the two *Acropora* species. In the following, by “fixed” difference we always mean fixed between *A. cervicornis* and *A. palmata*. It should be also be noted that such variants may be simply the result of demographic process rather than the result of adaptation to different niches.

Identified indels can also be analyzed to understand genomic difference between the studied species. Filtered in a manner analogous to the SNVs (requiring “high quality” and variability in *A. cervicornis* plus *A. palmata*), the original set of 940,345 genome-wide indels (Table 2) was reduced to 149,036. Of those, 2,031 were identified as fixed between *A. cervicornis* and *A. palmata*. They provide an additional set of hints for tracking down the genetic underpinnings of inter-species phenotypic differences, because indels are often more disruptive than substitutions.

### Examples of Substitutions with Potential Protein Modifications

We scanned the list of proteins with a fixed amino acid difference (or several fixed differences) to examine more closely. One potentially interesting fixed amino acid substitution is found in superoxide dismutase (SOD), whose activity is essential for almost any organism, and particularly for corals, like *Acropora*, that harbor symbionts of the genus *Symbiodinium* that generate superoxide radicals during photosynthesis (Dykens 1984; Lesser *et al.* 1990). This fixed difference was identified in comparison to *A. digitifera* (NCBI: LOC107335510 or Reef Genomic: Acropora_digitifera_12779), which strongly matched (E-value 3e-85) the human manganese SOD mitochondrial protein (GenBank: NP_001309746.1; Figure S4). We observed a glutamate (E) to glutamine (Q) substitution in *A. cervicornis*, corresponding to position 2 of the *A. digitifera* ortholog (Figure S4). According to the surveyed coral sequences, the Q is fixed in a number of other corals, except for an E shared by *A. digitifera*, *A. palmata*, *A. hyacinthus*, *A. millepora* and *A. tenuis* suggesting a lineage-specific reversion (Figure S4).

Another protein, NF-kappa-B inhibitor-interacting Ras-like protein 2 (NKIRAS2; NCBI: LOC107355568 and Reef Genomics: Acropora_digitifera_6635) has two putative fixed amino acid difference in the Caribbean acroporids (Figure S5). One, an E to aspartic acid (D) substitution, occurs in the middle of a “motif” LGT**E**RGV→LGT**D**RGV that is fairly well conserved between *A. palmata* and other members of the complex corals including *Porites* spp. and *Astreopora* sp. as well as robust corals except the Pocilloporidae family (*S. pistillata* and *Seriatopora spp*.), but not with *A. cervicornis* or other acroporids (Figure S5). Thus, this appears to be a recurrent substitution in acroporid and pocilloporid corals to ‘E’ with a reversion back to ‘D’ in A. *palmata*. The second putative fixed amino acid difference in this protein is unique to *A. cervicornis* from the corals we surveyed. The transition is from a polar but uncharged asparagine (N) to a positively charged lysine (K) in the short motif SVDGS**N**G→SVDGS**K**G (Figure S5). This substitution might have consequences on the tertiary structure and function of this protein in *A. cervicornis* compared to the other acroporids.

### Fixed Indels in Protein-Coding Regions

We also looked for fixed indels in protein-coding regions among corals compared to respective mammalian orthologs. Of the 2,031 fixed indels identified, most were not found in coding sequence with only 18 genes having a fixed indel. For closer inspection, we picked a fixed indel in STE20-related kinase adapter protein alpha (STRADα; NCBI: LOC107340566, Reef Genomics: Acropora_digitifera_13579) because it has a deletion of four-amino acids, along with two amino acid substitutions in *A. palmata*, both of which are fixed differences between the Caribbean acroporids (Figure S6). It aligns well with human STRADα, isoform 4 protein NP_001003788.1 (E-value 2e-77). A blastp search of coral resources indicated that the deletion is unique to *A. palmata* (Figure S6B), although *Madracis auretenra* also has a four amino acid deletion, but shifted by three positions. This deletion in *A. palmata* is confirmed by the lack of reads mapping to the 12bp nucleotide region (Figure S7).

To determine the degree of protein modification from these differences, we positioned them on a predicted protein structure of *A. cervicornis* using I-TASSER server (Yang *et al.* 2015). Figure S8 illustrates the predicted configuration of the protein using as structural reference the inactive STRADα protein annotated by Zeqiraj *et al.* (2009). The indel occurring between *A. palmata* and *A. cervicornis* is at positions 322 to 325, and the substitutions in positions 62 and 355. In order to induce the activation of STRADα, ATP binds and induces a conformational change. In its active stage, STRADα interacts with MO25α by means of the alpha-helixes B, C and E, the beta-laminae 4 and 5, and the activation loop to further regulate liver kinase B1 (LKB1) (Zeqiraj *et al.* 2009). Despite the fact that neither the substitutions nor the indel are placed in the structural elements described to interact with ATP or MO25α, it is difficult to disregard their functional role with them or with LKB1.

### KEGG Pathways Enriched for Fixed SNVs

**Figure 3:**
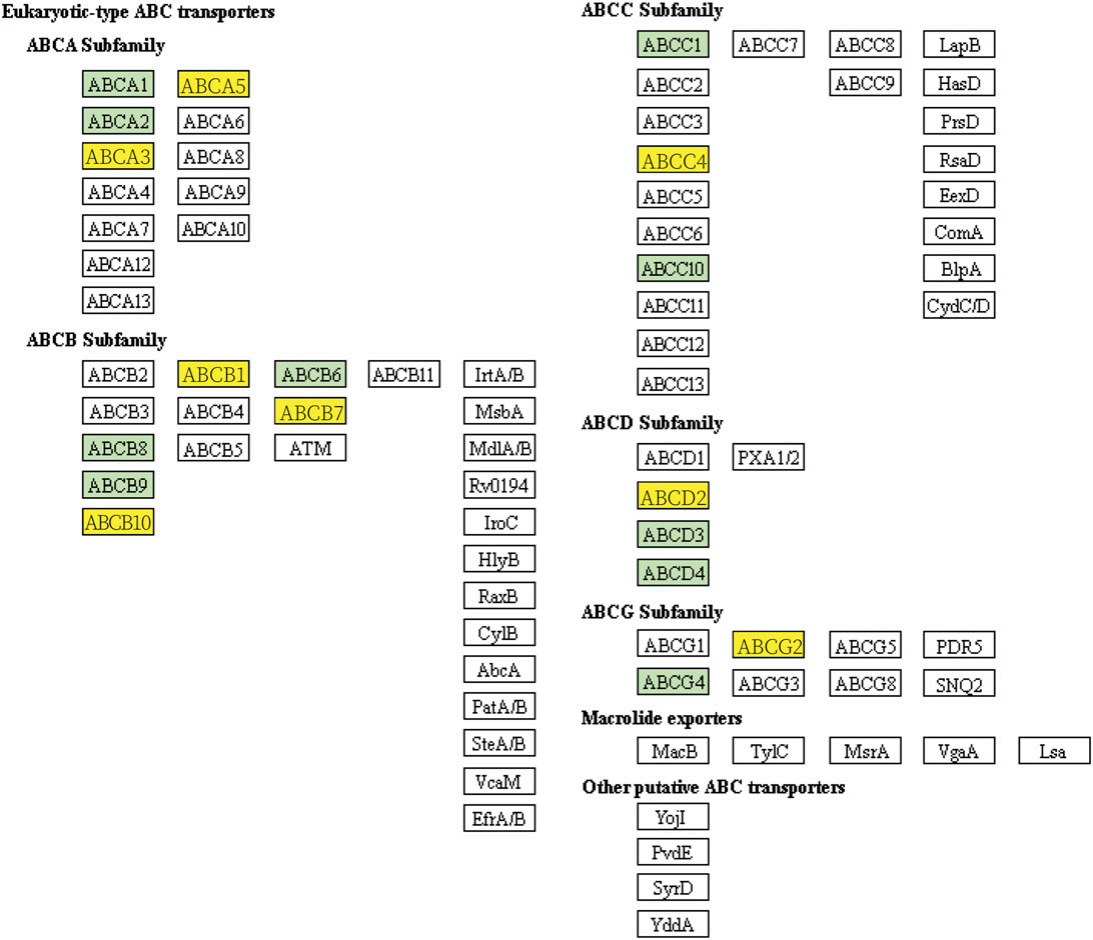
Pictorial representation of a portion of the KEGG pathway for ABC Transporters. The yellow shaded boxes indicate the genes having fixed amino acid differences between *A. cervicornis* and *A. palmata*. Green colored boxes indicate the genes that were found in these genomes but did not differ between the species. White colored boxes indicate the genes that were not found in the three acroporid genomes.

An alternative to looking at individual amino acid substitutions is to search for protein groupings that are enriched for substitutions. This is frequently done with Gene Ontology terms (Gene Ontology Consortium 2015) and/or classifications according to the KEGG (Kanehisa *et al.* 2017). We took advantage of the *A. digitifera* KEGG pathway annotations for 1,386 genes and looked for KEGG classes enriched for fixed amino acid variants. Five out of 119 pathways were found to be enriched in non-synonymous substitutions between *A. palmata* and *A. cervicornis* (two-tailed Fisher’s exact test, uncorrected *P* < 0.05), and included two pathways where up to 12 genes presented these differences (*i.e.*, ABC transporters and Wnt signaling pathway, Table 3). Following correction for false discovery rate, only the ABC transporters remained significantly enriched (FDR < 0.0022) with differences in 12 of 67 ABC transporters (Table 3). In particular, these 12 genes added 43 non-synonymous fixed differences between *A. palmata* and *A. cervicornis*, and were grouped into eight different KEGG modules (functional units represented by boxes, *i.e.*, ABCC4) within the pathway (Figure 3). Note that multiple proteins can be mapped to the same KEGG module (functional units represented by boxes, *i.e.*, ABCC4). Of these modules, ABCC4 grouped the largest number of genes (*n* = 5) and all the other modules had just one protein. Notably, the ABCB1 module included only one protein ortholog (NCBI: LOC107340542) that contains 20 non-synonymous fixed mutations between *A. palmata* and *A. cervicornis* (Figure S9). The majority of the substitutions are found in the cytoplasmic nucleotide binding domains (NBD), where ATP bind and hydrolysis occurs (Sauna and Ambudkar 2007). However, no changes were found in the conserved motifs that are proposed to form the ATP-binding pocket during NBD dimerization (Sauna and Ambudkar 2007).

**Table 3:** Statistically significant KEGG pathways enriched for genes having a fixed amino acid difference between *A. cervicornis* and *A. palmata*. The third column gives the number of genes in the pathway with one or more fixed difference(s), and the third reports what fraction they represent of all genes in the pathway. For instance, 67 of the genes are annotated as belonging to the ABC transporter pathway, and 12/67 = 0.18. Statistical significance determined using a two-tailed Fisher’s exact test. Notice that these *p*-values are not corrected for multiple tests. A randomization analysis indicates that adf02010 = ABC transporters is significant at a FDR of 0.0046. The number of predicted false positives are provided for each KEGG pathway.

Pathway	p-value	False Positives	# Genes	Fraction
**adf02010 = ABC transporters**	0.0015	< 1	12	0.18
**adf00790 = Folate biosynthesis**	0.019	1.13	5	0.21
**adf03420 = Nucleotide excision repair**	0.031	1.73	7	0.15
**adf04933 = AGE-RAGE signaling pathway in diabetic complications**	0.023	1.13	9	0.14
**adf04310 = Wnt signaling pathway**	0.037	2.42	12	0.12

Judged by the level of inter-species sequence conservation around the variant position, ABCD2 stands out. ABCD2 transports fatty acids and/or long chained fatty acyl-CoAs into the peroxisome (Andreoletti *et al.* 2017). The variant valine (V) appears to at the beginning of transmembrane helices 3 that is conserved in the majority of coral species, including *A. digitifera* and *A. cervicornis* (Figure S10). In *A. palmata* and *A. millepora* the V is replaced by isoleucine (I). However, the residues predicted to stabilize ABCD proteins and facilitate transport across the membrane are conserved between all corals and the human ortholog (Andreoletti *et al.* 2017). In vertebrates, the “motif” SVAHLYSNLTKPILDV is essentially conserved in all mammal, bird, and fish genomes available at the UCSC browser (Figure S11). The only three substitutions pictured in Figure S11 are a somewhat distant I→V in hedgehog and rabbit, and V→I in opossum at the position variant in *A. palmata* and *A. millepora*. This extreme level of inter-species protein conservation suggests that the ABCD2 ortholog may function somewhat differently in *A. palmata* and *A. millepora* compared to most other corals. However, the ease with which V and I can be interchanged in nature, because of their biochemical similarity and illustrated by the mammalian substitutions mentioned above, tempers our confidence in this prediction. Still, the apparent near-complete conservation of this particular valine in evolutionary history lends some weight to the hypothesis.

### Genomic Stretches of SNVs

**Figure 4:**
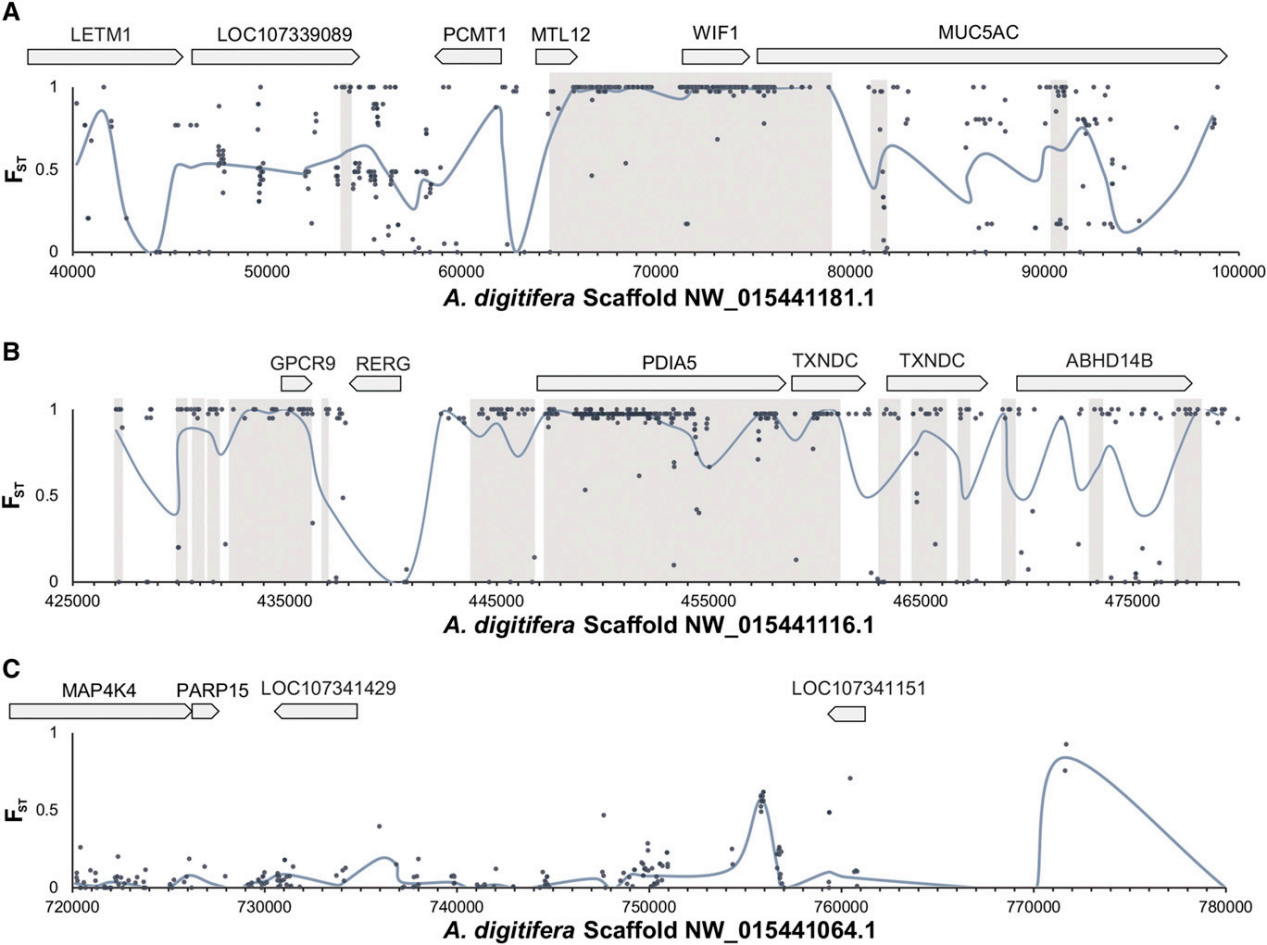
Genomic intervals with or without regions of differentiation between *A. palmata* and *A. cervicornis*. Inter-species allelic differentiation (*F*_ST_) was calculated using the unbiased Reich-Patterson estimator (Reich *et al.* 2009). High scoring regions are shaded in light gray along 60 kb genomic windows for the top two scoring intervals, scaffold NW_015441181.1 (A) and scaffold NW_015441116.1 (B), compared to 60 kb genomic window on scaffold NW_015441064.1 with no intervals (C). Gray points are the *F*_ST_ estimate for each SNVs and blue line is the average *F*_ST_ calculated over 1 kb sliding window analysis. Predicted genes within these windows are shown above the graph in gray arrows. In order, genes include *mitochondrial proton/calcium exchanger protein (LETM1)*, *A. digitifera LOC107339089, protein-L-isoaspartate (D-aspartate) O-methyltransferase (PCMT1), mitochondrial methyltransferase-like protein 12 (MTL12), Wnt inhibitory factor 1 (WIF1), mucin-5AC-like (MUC5AC), G protein-coupled receptor 9 (GPCR9), Ras-related and estrogen-regulated growth inhibitor (RERG), protein disulfide-isomerase A5 (PDIA5), thioredoxin domain containing protein (TXNDC), protein ABHD14B (ABHD14B), mitogen-activated protein kinase kinase kinase kinase 4 (MAP4K4), poly(ADP-ribose) polymerase family member 15 (PARP15)*, *A. digitifera LOC107341429, and A. digitifera LOC107341151*.

**Figure 5:**
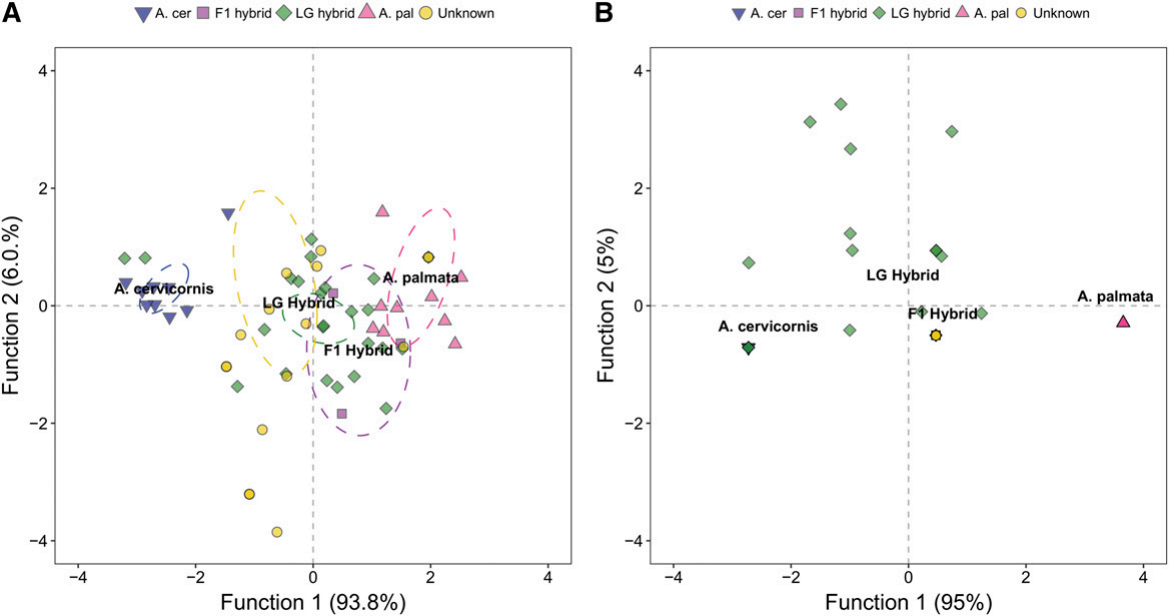
Discriminant factorial correspondence analysis of five microsatellite markers (A) and eight species-specific SNV loci (B). Samples were assigned to four different groups based on their previous taxon assignment: 1. *A. cervicornis* (*n* = 9, blue upside down triangles), 2. *A. palmata* (*n* = 10, pink triangles), 3. F1 hybrids (*n* = 3, purple squares), and 4. later generation hybrids (*n* = 24, green diamonds). The remaining hybrid samples (*n* = 20, yellow circles) had no previous hybrid assignment and acted as our test set for the analysis. In panel B, multiple data points for pure bred colonies are not visible because their coordinates are identical. F1 hybrids, test hybrids and seven later generation hybrids are also masked as they share the same coordinates as the F1s, representing F1-like hybrids in the data set.

Rather than restricting the analyses to only the fixed SNVs, a larger set of the high-quality SNVs related to the species differences can be identified by interrogating the joint allele-frequency spectrum of the two species. An advantage of this approach over considering just amino acid variants is that it can potentially detect functional changes in non-coding regions, such as promoters or enhancers. We identified 12,279 intervals of consecutive SNVs with high *F*_ST_ values, sometimes referred to as “genomic islands of divergence” (Nosil *et al.* 2009). The genomic intervals ranged in size from 5 b (NW_015441140.1:321,729-321,734, 4 SNVs with average *F*_ST_ = 1.0) to 27 kb (NW_015441096.1: 814,882-842,464, 8 SNVs with average *F*_ST_ = 0.9217) and contained 5.7% of all SNVs (96,594 out of 1.69 million SNVs). A subset of these islands with high *F*_ST_ values also has significantly lower nucleotide diversity (π) within a species and higher absolute divergence (d_XY_) between the species than the genomic background based on a genomic scan of 500 bp windows (*n*= 2,552 intervals, Mann Whitney *U*-test, *P* < 2e-16, Figure S12). This is consistent of two divergent species with little gene flow. The top scoring interval covers a 14 kb window in positions 64,603-78,897 of scaffold NW_015441181.1 (Table S5 and Figure 5A). The average *F*_ST_ for the 241 SNVs in this interval is 0.9821, while the average *F*_ST_ for all of the roughly 1.7 million SNVs is 0.2719 (Figure S12C). Within this interval, there are three gene models: *methyltransferase-like protein 12* (*MTL12*; NCBI: LOC107339088), *Wnt inhibitory factor 1-like protein* (*WIF1*; NCBI: LOC107339060), *mucin-5AC-like protein* (*MUC5AC*; NCBI: LOC107339062) (Figure 4A).

The next highest scoring run of high *F*_ST_ values is the 15 kb interval in positions 447289-462570 of scaffold NW_015441116.1 (Figure 4B). The 306 SNVs in this region have an average *F*_ST_ = 0.9756. The most recent NCBI gene annotations mention two intersecting genes in the interval, *protein disulfide-isomerase A5-like* (*PDIA5*; NCBI: LOC107334364), mapping to the interval 447,296-458,717, and *thioredoxin domain-containing protein 12-like* (*TXNDC12*; NCBI: LOC107334366), mapping to 459,123-462,401 (Table S5 and Figure 4B). Adjacent to this interval are three lower scoring intervals also containing a gene annotated as *TXNDC12* (NCBI: LOC107334421), mapping to 463,276-467,160 (Table S5 and Figure 4B).

The mapping of LOC107334366 shows a strong match to seven exons, but the mapping of LOC107334364 include weakly aligning exons and missing splice signals. LOC107334364 consists of three weakly conserved tandem repeats, and has partial blastn alignments to position 33-172 of human thioredoxin domain-containing protein 12 precursor (GenBank: NP_056997.1). The shorter sequence LOC107334366 has a blastp alignment (E-value 9e-22) to the same region. In the older Reef Genomics dataset for *A. digitifera*, the corresponding gene for LOC107334364 was Acropora_digitifera_14046l. Thus, based on the newer NCBI annotation, there appears to be either a gene or a pseudo-gene in this highly divergent genomic region of *A. digitifera*.

### SNV Markers for Species Identification and Hybrid Assignment

To aid the design of genotyping studies we identified 894 “PCR-ready” SNVs as those that do not have another SNV, indel, or any (interspersed or tandemly duplicated) repeats within 50 bp (Table 2). We call these the “PCR-ready” SNVs, because in theory they are good candidates for amplification in any of the three *Acropora* species. We validated a subset of eight of these PCR-ready SNVs in additional *A. palmata* (*n* = 10) and *A. cervicornis* (*n* = 9) samples from across the geographic range (Table S2) using a RFLP assay. The eight markers were designed to digest the PCR product at a single nucleotide base present in only one of the two species (Table S3). For example, at locus NW_015441435.1 position 299429, the variable base between the species (GG in *A. cervicornis* and AA in *A. palmata*) provides a unique recognition site in *A. cervicornis* for the restriction enzyme *Hpy*CH4IV (A^CG_T) that results in digestion of *A. cervicornis* PCR product but not *A. palmata* (Figure S13A). We found that our stringent selection of PCR-ready SNVs are in fact fixed in the additional samples surveyed.

We also screened colonies that were morphologically classified as hybrids between *A. palmata* and *A. cervicornis*. We attempted to refine the hybrid classification of colonies into first or later generation hybrid groups (putative backcrosses and F2s) based on the proportion of ancestry from each parental species using five microsatellite markers or the above described eight SNV loci.

Using the SNV markers, the reference F1 hybrids and seven later generation hybrids were heterozygous at all variable sites, whereas the remaining later generation hybrids (*n* = 17) genotypes at each site varied depending on the locus (two examples in Figure S13). Similar to the F1 hybrids, the test set of hybrids were also heterozygous at all loci.

The congruency of taxon classification was compared between the SNV multi-locus genotypes (MLGs) and microsatellite MLGs using a discriminant factorial correspondence analysis (DFCA) for each marker set (Figure 5). All *A. cervicornis* samples were correctly identified to their taxonomic group using the microsatellite MLGs, but in only 60% of *A. palmata* colonies did the microsatellite clustering coincide with the previous taxon assignment (Table S2 and Figure 5A). In contrast, because of stringency in selecting the fixed SNV loci, there was 100% agreement of the previous taxon assignment of the parental species colony and its SNV MLG classification (thus data points for pure bred samples are overlaid by the group centroid in Figure 5B).

No hybrid samples (F1, later generation or those in the test set) were assigned with high probability to the F1 group with either maker set in the DFCA (Table S2). However, we found that the SNV MLGs of F1 hybrids, seven later generation hybrids and all test hybrids shared the same discriminant function coordinates as the F1 centroid, representing F1-like hybrids in the data set (overlaid by F1 group centroid in Figure 5B). The remaining later generation hybrids were classified as either *A. cervicornis* (*n* = 5) or hybrid (*n*= 12; Figure 5B and Table S2).

## Discussion

In this study, we have identified inter- and intra-species SNVs and indels between three *Acropora* species. These variants can cause amino acid substitutions that might ultimately alter protein function between these corals. We provided examples of genes with putative fixed-differences between the Caribbean acroporid species, grouped variants by their KEGG pathways, highlighting examples from the ABC transporter pathways, identified highly diverged genomic regions between them and developed a RFLP assay to distinguish species and hybrids. Genomic resources and workflows are available on Galaxy allowing researchers to reproduce the analyses in this paper and apply them to any acroporid species or other non-model organisms.

### Candidate Loci for Microbe Interactions and Cellular Stress

We highlighted several genes with fixed differences between the two Caribbean acroporids that are involved in innate immunity, membrane transport and oxidative stress in cnidarians. These genes are also important for mediating interactions between the coral host and their microbial symbionts. Corals mediate interactions with foreign microbes by either creating physical barriers or initiating an innate immune response (Palmer and Traylor-Knowles 2012; Oren *et al.* 2013). Innate immunity is not only activated for the removal of threatening microbes, but also facilitates colonization of beneficial microorganisms within the coral host.

As one of the physical barriers, corals secrete a viscous mucus on the surface of their epithelium that can trap beneficial and pathogenic microbes (Sorokin 1973; Rohwer *et al.* 2002). Microbial fauna of the mucus can form another line of defense for their host, with evidence that mucus from healthy *A. palmata* inhibits growth of other invading microbes and contributes to the coral antimicrobial activity (Ritchie 2006). This mucus is composed of mucins, one of which might be *mucin 5AC* that was found to span three divergent genomic intervals between *A. palmata* and *A. cervicornis*. Mucin-like proteins have been found in the skeletal organic matrix of *A. millepora* (Ramos-Silva *et al.* 2014) and are differentially expressed in the tips of *A. cervicornis* during the day (Hemond and Vollmer 2015) suggesting a potential role for these large glycoproteins in biomineralization as well. Thus, the divergence of mucin protein in elkhorn and staghorn corals could underlie difference in the composition of their mucus and/or calcification patterns.

Beyond the mucus layer, corals and other cnidarians have a repertoire of innate immune tools to recognize microbial partners from pathogens and remove the latter. The transcription factor NF- κB is one of these tools that regulates expression of immune effector genes, including mucin mentioned above (Sikder *et al.* 2014). We identified two fixed SNVs in NKIRAS2, an inhibitor of NF-κB transcription (Chen *et al.* 2004). The two substitutions within this protein were both unique to either *A. palmata* or *A. cervicornis* and neither were shared by the Pacific acroporids. While the role of NKIRAS1 and -2 are largely unexplored in non-mammal animals, *NKIRAS1* has been reported to be one out of nine genes down-regulated at high temperatures in *A. palmata* (Polato *et al.* 2013).

Another candidate protein STRADα (Figure 3) is part of the AMP-activated protein kinase (AMPK) pathway, which plays a key role in cellular growth, polarity and metabolism. Under starvation or stressful conditions, the AMPK pathway senses cell energy and triggers a response to inhibit cell proliferation and autophagy (Hawley *et al.* 2003). Recently, the switch toward activation of AMPK-induced autophagy over apoptosis has been proposed to enhance disease tolerance in immune stimulated corals (Fuess *et al.* 2017). In this study, STRADα was found to have two non-synonymous mutations and an indel between *A. cervicornis* and *A. palmata* (Figure S8). Although these changes do not occur in a reported site of activity, we cannot ignore the possibility that they are relevant in the interaction of STRADα with MO25Aα and LKB1. The products of these three proteins interact together to regulate the AMPK cascade, with STRADα being key for LKB1 protein stability. The extent to which AMPK more broadly contributes to the development and disease tolerance of elkhorn and staghorn corals needs to be further explored.

As a way to interact and exchange nutrients with their beneficial microbes, corals can use ABC transporter proteins. In general, ABC transporters encode for large membrane proteins that can transport different compounds against a concentration gradient using ATP. More specifically, they can transport long-chain fatty acids, enzymes, peptides, lipids, metals, mineral and organic ions, and nitrate. ABC transporters were enriched in fixed amino acid differences between *A. palmata* and *A. cervicornis* (Figure 3). Previous characterization of the proteins embedded in a sea anemone symbiosome, the compartment where the symbionts are housed, found one ABC transporter which could facilitate movement of molecules between partners (Peng *et al.* 2010). Further evidence for metabolite translocation via ABC transporters comes from the enrichment of genes, including *ABCD2*, and associated metabolites in the colonization of a sea anemone with a heterologous symbiont *Durusdinium trenchii* (Matthews *et al.* 2017). ABC transporters were also upregulated in response to high CO_2_ concentrations (Kaniewska *et al.* 2012) and during the day (Bertucci *et al.* 2015) in *A. millepora* suggesting diverse roles for these proteins, transporting both molecules from the environment and metabolites from their symbionts. Within the ABC transporters, we analyzed in detail the non-synonymous mutations in ABCB1 and ABCD2 between *A. palmata* and *A. cervicornis* (Figure S9 and Figure S10). The ABCB1 group encodes p-glycoproteins that are important for the efflux of toxic compounds from the cells. *ABCB1* gene expression changes with heavy metal exposure in the sea anemone *Nematostella vectensis* (Elran *et al.* 2014) and the coral *Orbicella franksi* (Venn *et al.* 2009), and protein abundance increases with local anthropogenic stressors in *Orbciella annularis* (Downs *et al.* 2005). The abundance of mutations in the nucleotide binding domains (NBDs, Figure S9) between *A. palmata* and *A. cervicornis* is consistent with observations of higher substitution in less evolutionarily conserved sites in the NBDs of ABCB1 between 11 eukaryotic species, including functionally characterized SNVs associated with variation in human drug response (Wolf *et al.* 2011). The consequences of 20 non-synonomous substitutions on the function and substrate specificity of this protein between the two species remains unclear and should be investigated further.

The analysis for ABCD2 was limited by the availability of sequences, but allowed us to conclude that the amino acid substitution, though expected to not produce a large functional change, is embedded in a well-conserved motif. The ABCD2 product is involved in the transport of very long-chain acyl-CoA into peroxisomes for β-oxidation. It has been reported that *A. palmata* larvae derive their energy by this mean and that high temperatures induce a change in expression of genes associated with peroxisomal β-oxidation (Polato *et al.* 2013). This is thought to indicate that larvae of *A. palmata* catabolize their lipid stores more rapidly at elevated temperatures (Polato *et al.* 2013). Increased lipid catabolism in turn drove the need for additional redox homeostasis proteins to deal with reactive oxygen species (ROS) produced during oxidation of fatty acids (Polato *et al.* 2013).

Superoxide dismutase, PDIA5 and TXDNC12 are involved in ROS stress-response and antioxidant defense to deal with the oxygen radicals that are produce via the coral host or its symbionts. It has been reported that the antioxidant protein SOD, which converts superoxide anions to hydrogen peroxide, is important to reduce the ROS produced by the coral host and also its dinoflagellate symbiont (Levy *et al.* 2006), particularly under high temperature stress (Downs *et al.* 2002), high photosynthetically active radiation (Downs *et al.* 2002) and salinity stress (Gardner *et al.* 2016). The genes *PDIA5 and TXDNC12* also regulate oxidative stress as well as protein folding. They are both localized to the endoplasmic reticulum and belong to the thioredoxin superfamily of proteins (Galligan and Petersen 2012). These genes were found to span the longest interval of significant genomic differentiation between the two Caribbean species (Figure 5). Thioredoxin-like genes have been differentially expressed in a number of thermal stress experiments on Pacific acroporids (Starcevic *et al.* 2010; Souter *et al.* 2011; Rosic *et al.* 2014) providing strong support for their role in mediating redox stress. Future research is required to validate the functional consequences of the substitutions in the loci that differ between *A. palmata* and *A. cervicornis* and their putative roles in host cellular stress response, microbial interactions and/or nutrient exchange.

### Mitochondrial SNVs

Unlike other metazoan mitochondrial DNA (mtDNA), cnidarian mtDNA evolves much slower and is almost invariant among conspecifics (van Oppen *et al.* 1999; Shearer *et al.* 2002). However, the so-called control region can be hypervariable compared to the other mtDNA regions in corals (Shearer *et al.* 2002), and is where the majority of the mitochondrial SNVs in these taxa were identified (Figure S3). The variability in this gene-free region has been used in previous studies to reconstruct the phylogenetic relationship of all acroporid species (van Oppen *et al.* 2001) and as one of the markers to determine gene-flow between *A. palmata* and *A. cervicornis* from hybridization (Vollmer and Palumbi 2002, 2007). The lack of fixed-differences between the mtDNA of these two species suggests that mito-nuclear conflict might be limited or non-existent during hybridization of these species. In the future, these mtDNA markers might resolve interesting patterns about mitochondrial inheritance and evolutionary relationships between the acroporids.

### Species-Specific Diagnostic Markers

We validated eight of the PCR-ready fixed SNVs in additional acroporid samples and classified the two acroporid species and their hybrid based on the MLGs of these makers and five microsatellite loci (Figure 5). Currently, microsatellite makers are routinely used to identify acroporid genotypes and clone mates, but only one of these is a species-specific marker (locus 192) between the Caribbean acroporids (Baums *et al.* 2005; Baums *et al.* 2009). While previous studies have used one mitochondrial and three nuclear loci to study Caribbean hybrid *Acropora* (Oppen *et al.* 2000; Vollmer and Palumbi 2002), PCR-ready fixed SNV markers provide an alternative for high-throughput genotyping and hybrid classification. The detection of only one variable base at each SNV locus can lower genotyping error, avoid difficulties in interpreting heterozygous Sanger sequences and increase reproducibility across labs (Anderson and Garza 2006). Our results indicate a small number of fixed SNVs can outperform the microsatellite makers for taxonomic classification of the species but not necessarily the hybrids. Our inability to discriminate the F1-like hybrids from the later generation hybrids with the DFCA is likely due to the low sample size of reference F1 hybrids (*n* = 3). In the case of the SNV markers, the identical MLGs between the F1 hybrids and seven later generation hybrids further reduced our ability to separate the groups. Therefore, with the limited number of PCR-ready SNVs tested, there was no difference in the performance of microsatellite to SNV loci for refining hybrid classification. These results, however, indicate that the genomes provide a rich source for PCR-ready SNVs, albeit a larger number of SNVs than tested here will need to be assayed before Caribbean acroporid hybrids can be classified confidently.

### Conclusion

By using the genome assembly of *A. digitifera*, we were able to detect differences between *A. cervicornis* and *A. palmata* at various levels, from a single nucleotide substitution to hundreds of nucleotide substitutions over large genomic intervals. We identified genetic differences in key pathways and genes known to be important in the animals’ response to the environmental disturbances and larval development. This project can work as a pilot to gather intra- and interspecies differences between *A. cervicornis* and *A. palmata* across their geographic range. Ultimately, gene knock-down and gene editing experiments are needed to test whether these and other genetic differences have functional consequences and thus could be targets for improving temperature tolerance and growth of corals.
